# Health-Related Quality of Life in Breast Cancer Patients Undergoing Chemotherapy: A Cross-Sectional Study in Greece

**DOI:** 10.3390/medicina62061196

**Published:** 2026-06-21

**Authors:** Anastasia Karagiannaki, Vasiliki Michou, Evangelia Antoniou, Menelaos Zafrakas, Panagiotis Eskitzis

**Affiliations:** 1 Department of Midwifery, School of Healthcare Sciences, University of Western Macedonia, Keptse, 50200 Ptolemaida, Greece; anastasiakaragiannaki@hotmail.com (A.K.); peskitzis@uowm.gr (P.E.); 2 Department of Nursing, School of Health Sciences, International Hellenic University, 57400 Thessaloniki, Greece; 3 Department of Midwifery, University of West Attica, 12243 Athens, Greece; lilanton@uniwa.gr; 4 Department of Midwifery, School of Health Sciences, International Hellenic University, 57400 Thessaloniki, Greece

**Keywords:** breast cancer, health-related quality of life, HRQoL, EORTC QLQ-C30, clinical characteristics, sociodemographic factors

## Abstract

*Background and Objectives:* Quality of life (QoL) is an important issue for breast cancer (BC) survivors. The objective of this study was to assess health-related QoL (HRQoL) of BC patients and investigate the impact of different demographic and clinical factors on physical and social functioning and BC-related symptoms. *Materials and Methods:* In this cross-sectional study, 107 BC patients undergoing chemotherapy in Greece completed a questionnaire collecting sociodemographic and clinical information and the European Organization for Research and Treatment of Cancer Quality of Life Questionnaire–Core 30 (EORTC QLQ-C30) in order to assess HRQoL. Descriptive statistics and multiple linear regression analyses were used to identify factors linked to HRQoL outcomes. *Results:* Overall, participants reported moderate HRQoL, with high physical and social functioning and moderate emotional, cognitive, and role functioning. Fatigue was the most common symptom, whereas other symptoms were generally uncommon. Multiple regression analyses showed that marital status, place of residence, time since diagnosis, and type of surgery were significantly associated with the global QLQ-C30 score (R^2^ = 0.337, *p* < 0.001). Physical functioning was associated with comorbidity burden, time since diagnosis, and employment status (R^2^ = 0.155, *p* = 0.035), and social functioning with marital status and type of surgery (R^2^ = 0.171, *p* = 0.011). Emotional functioning showed exploratory associations with place of residence and type of surgery; however, the overall regression model for emotional functioning did not reach statistical significance. No symptom model reached overall significance, but time since diagnosis, treatment type, and surgery were linked to distinct symptoms. *Conclusions:* BC patients undergoing chemotherapy in Greece report an overall moderate level of HRQoL, which is significantly influenced by a combination of demographic and clinical factors; physical and social functioning were high, with moderate emotional, cognitive, and role functioning. These findings highlight the importance of individualized supportive care strategies in order to improve QoL of BC patients.

## 1. Introduction

Breast cancer (BC) remains one of the most frequently diagnosed malignancies worldwide and a major public health challenge [1,2]. According to the American Cancer Society, BC incidence has increased by approximately 1% annually since 2012, particularly among women younger than 50 years and Hispanic and Asian American/Pacific Islander populations [3,4]. In the United States, an estimated 316,950 new BC cases and 42,170 BC-related deaths were expected among women in 2025 [3]. Furthermore, cancer statistics projected for 2026 in the United States indicate that BC will continue to be the leading cancer diagnosis among women, representing nearly one-third of all newly diagnosed cases. Along with lung and colorectal cancer, it accounts for approximately half of all incident cancers in the female population [5]. This global trend is also evident across Europe. According to the International Agency for Research on Cancer (IARC) of the World Health Organization, it has been estimated that 557,873 new cases of female BC were diagnosed in Europe in 2022, making it the most frequently diagnosed cancer among women in the region. The distribution of these cases reveals significant variation between European countries. The Russian Federation recorded the largest number of cases, with 78,839 (14.1%), followed by Germany with 74,016 (13.3%), France with 65,659 (11.8%), the United Kingdom with 58,756 (10.5%), and Italy with 57,480 (10.3%) cases. The remaining European countries accounted for approximately one-third of all cases, reflecting considerable regional variation [6]. In Greece, BC represents the most frequently diagnosed malignancy when both sexes are considered, with an estimated age-standardized incidence rate (world standard) of 258.7 per 100,000 persons in 2022, highlighting its dominant contribution to the national cancer burden [6]. These statistics highlight the persistent disparities in BC burden across Europe and underscore the urgent need for region-specific strategies aimed at improving prevention, early detection, and access to care.

The quality of life (QoL) of BC survivors is a complex concept encompassing physical, psychological, social, and functional well-being [7,8]. BC and its treatments, such as surgery, cytotoxic chemotherapy and targeted therapy, radiotherapy, and hormonal therapy, often lead to issues like fatigue, pain, menopausal symptoms, body image disturbances, and psychological distress [9]. These effects can significantly impact daily activities and emotional health. Research shows that QoL is affected by important socio-demographic and clinical factors, including age, marital status, educational level, place of residence, treatment modalities, and time since diagnosis. Understanding these factors is crucial for assessing and enhancing QoL outcomes for women with BC [9,10,11,12,13].

In Greece, limited research has examined the health-related quality of life (HRQoL) of women with BC [12], particularly during active treatment phases such as chemotherapy. Moreover, existing studies often focus on specific clinical stages or treatment periods and provide inconsistent findings regarding the role of demographic and clinical determinants of HRQoL. Therefore, further evidence is needed to better understand how these factors interact and influence quality of life in this population. The present cross-sectional study aimed to assess HRQoL among women with BC undergoing chemotherapy in Greece and to investigate the influence of selected sociodemographic and clinical factors on functional domains and symptom burden using the European Organization for Research and Treatment of Cancer Quality of Life Questionnaire—Core 30 (EORTC QLQ-C30). By examining multiple demographic and treatment-related variables simultaneously, this study seeks to provide additional evidence on the determinants of HRQoL in Greek BC patients and to inform individualized supportive care strategies.

## 2. Materials and Methods

### 2.1. Participants

Female BC patients who expressed willingness to participate were consecutively screened for eligibility according to the following predefined inclusion and exclusion criteria. Inclusion criteria were age ≥ 18 years and current chemotherapy treatment for BC. Exclusion criteria included age < 18 years, BC treatment not yet initiated at the time of recruitment, prior participation in a similar study, and refusal or inability to provide the required study information. Recruitment was conducted over a six-month period, from May to October 2023.

### 2.2. Sample Size Estimation

Sample size estimation was conducted using G*Power software (version 3.1). The selected effect size (≥0.15) corresponded to a moderate effect according to Cohen’s criteria for multiple regression analyses [14] and was considered appropriate for detecting relevant associations in HRQoL outcomes. Similar moderate effect sizes have previously been reported and interpreted as clinically meaningful in HRQoL studies involving breast cancer patients and EORTC QLQ-C30 outcomes [15,16]. Assuming a medium effect size (f^2^ = 0.21), a significance level of α = 0.05, and a statistical power of 80% for the planned multiple regression analyses, the minimum required sample size was estimated at 84 participants. According to Cohen’s guidelines, effect sizes for regression analyses are categorized as small (f^2^ = 0.02), medium (f^2^ = 0.15), and large (f^2^ = 0.35), indicating that the selected effect size corresponds to a moderate-to-large effect [14].

### 2.3. Study Design

This cross-sectional study was conducted among women diagnosed and treated for BC at Hippokration General Hospital in Thessaloniki, Greece, who were invited to participate. Participants who voluntarily agreed to take part in the study were required to complete two questionnaires: (1) a general questionnaire collecting demographic and clinical information related to BC and (2) the Greek version of the European Organization for Research and Treatment of Cancer Quality of Life Questionnaire—Core 30 (EORTC QLQ-C30) in order to assess quality of life (QoL).

### 2.4. Minimization of Potential Bias

To minimize bias in accordance with STROBE recommendations, several measures were implemented. Information bias was reduced using the validated Greek version of the EORTC QLQ-C30 questionnaire [13], combined with identical written instructions and similar conditions for all participants. Selection bias was addressed by consecutively recruiting eligible patients according to the aforementioned predefined inclusion criteria. Consistent application of these criteria and standardized data collection procedures ensured uniformity across participants. Additionally, relevant demographic and clinical variables were included in multivariable linear regression analyses to adjust for potential confounding effects on HRQoL outcomes.

### 2.5. Data Collection and Verification

Missing data were assessed prior to statistical analysis. No missing data were identified, as all questionnaires were administered and completed under the supervision of the primary investigator (A.K.), who directly collected patients’ responses. Patients completed the questionnaires in the hospital wards where they were admitted or in the outpatient clinics of Hippokrateion General Hospital, Thessaloniki, Greece. Completed questionnaires were immediately reviewed for completeness and consistency, and any unclear or ambiguous responses were clarified directly with participants on site. Next, all data were double-entered and cross-checked against the original forms to ensure accuracy and minimize entry errors. Consequently, all analyses were conducted using complete datasets, and no imputation methods were required.

### 2.6. General Questions Regarding Demographic and Other Personal Information

Participants completed a comprehensive questionnaire in Greek designed to collect demographic and clinically relevant data, including age, smoking status, any comorbidities present, ethnicity, marital status, educational level, residential location, and type of surgical and medical treatment for BC. An English version of this questionnaire is provided in Supplementary Material, Supplementary SA.

### 2.7. EORTC QLG Core Questionnaire (EORTC QLQ-C30)

The EORTC QLQ-C30 is a 30-item questionnaire designed to assess the functional status and symptom burden that impact the QoL of individuals with cancer (see Supplementary Material, Supplementary SB, Table S1). It consists of three main components:

- The Global Health Status and QoL (GHS) scale.
- Functional scales, which evaluate five areas: physical functioning, role functioning, emotional functioning, cognitive functioning, and social functioning.
- Symptom scales, which measure various issues, including fatigue, nausea and vomiting, pain, dyspnea, insomnia, loss of appetite, constipation, diarrhea, and financial difficulties caused by the patient’s physical condition or medical treatment.

According to the EORTC QLQ-C30 scoring manual (version 3.0), the functional and symptom scales are derived from specific questionnaire items as follows: physical functioning (items 1–5), role functioning (items 6–7), emotional functioning (items 21–24), cognitive functioning (items 20 and 25), and social functioning (items 26–27). The Global Health Status/QoL scale comprises items 29–30. The multi-item symptom scales include fatigue (items 10, 12, 18), nausea and vomiting (items 14–15), and pain (items 9, 19). Single-item symptom measures include dyspnea (item 8), insomnia (item 11), loss of appetite (item 13), constipation (item 16), diarrhea (item 17), and financial difficulties caused by the patient’s physical condition or medical treatment (item 28). All scores were linearly transformed to a 0–100 scale according to the EORTC scoring manual.

Generally, higher scores on the functional scales indicate better functioning, while higher scores on the Global Health Status/QoL scale suggest better overall QoL. Conversely, higher symptom scale scores indicate greater severity of symptoms or related issues. In this study, the validated Greek version (version 3.0) of the QLQ-C30 questionnaire was utilized [17]. The QLQ-C30 has been previously validated for use in Greek BC patients (Cronbach’s α = 0.86) [18] and individuals with multiple myeloma (Cronbach’s α > 0.70) [19], demonstrating its reliability as a QoL assessment tool for the Greek population.

### 2.8. Confounding Factors

The identification and management of potential confounding variables were based on theoretical considerations and existing evidence regarding HRQoL in women with BC. Key confounders included age, marital status, educational level, place of residence, comorbidity burden, BC history, type of surgery, and current treatment characteristics. To account for these confounders, a multivariable linear regression analysis was conducted using the overall QLQ-C30 score and selected functional domain scores as dependent variables. This approach allowed assessment of the independent associations between key predictors and HRQoL outcomes while accounting for the influence of measured confounders.

### 2.9. Ethical Approval

For the preparation of this study, a comprehensive research protocol, relevant study information, and the informed consent form (Supplementary Material, Supplementary SC) were submitted to the Research Ethics Committee of the University of Western Macedonia, Greece, and ethical approval was granted on 31 March 2023 (Protocol No.: 178/2023). The study proposal provided a detailed description of the research objectives, methodological framework, confidentiality measures, and anonymity safeguards, in accordance with the General Data Protection Regulation (GDPR), European Union Regulation (EU) 2016/679, and the Declaration of Helsinki. In addition, approval to conduct the study at Hippokration General Hospital in Thessaloniki, Greece, was granted by the Hospital Committee for Quality Assurance of Research and Continuing Education (approval no. 23794, 22 May 2023).

### 2.10. Statistical Analysis

Statistical analysis was conducted using the IBM Statistical Package for Social Sciences (IBM SPSS Statistics for Windows, Version 28.0; IBM Corp., Armonk, NY, USA, Released 2022). The normality of variable distributions was assessed using the Kolmogorov–Smirnov test. Demographic and clinical data results for normally distributed quantitative variables are presented as means ± standard deviation, while categorical variables are expressed as frequencies (n) and percentages (%). For the analysis of the QLQ-C30 subscales, data were presented as the mean (M) ± standard deviation (SD), along with the 95% confidence interval (lower/upper bound) and minimum (Min) and maximum (Max) values for each subscale. Group comparisons for continuous outcomes (QLQ-C30 subscales scores) were performed using independent *t*-tests for dichotomous variables (e.g., comorbidities: Yes/No) and one-way ANOVA for variables with three or more categories (e.g., type of surgery). Lastly, multiple linear regression analyses were conducted to evaluate the influence of potential confounding variables on overall QLQ-C30 scores and their individual subscales. Prior to interpretation of the regression models, multicollinearity was assessed using variance inflation factor (VIF) and tolerance statistics. No evidence of problematic multicollinearity was identified, with acceptable VIF and tolerance values observed across all regression models. In addition, regression assumptions were evaluated through residual diagnostics. Visual inspection of histograms and normal probability (P–P) plots of standardized residuals indicated an approximately normal distribution of residuals. Moreover, scatterplots of standardized residuals against standardized predicted values showed no discernible patterns or funnel-shaped distributions, suggesting homoscedasticity. Standardized residuals ranged from −2.41 to 2.50, indicating the absence of problematic outliers. Overall, these diagnostic assessments supported the suitability of the linear regression models employed in this study. Since multiple univariate comparisons were performed across several HRQoL domains, these analyses should be considered exploratory and interpreted with caution, given the increased risk of type I error. Statistical significance was set at *p* ≤ 0.05.

## 3. Results

### 3.1. Patient Demographic and Clinical Data

A total of 107 women with BC voluntarily provided informed consent to participate and were included in the present study. Their demographic and clinical characteristics of patients are presented in Table 1.

### 3.2. EORTC QLQ-C30 Results

The descriptive analysis results for each item of the QLQ-C30 scale are shown in Supplementary Table S1 (Supplementary SB). The descriptive analysis of the subdimensions of QoL among the female participants revealed that their overall health status was at a moderate level (M = 51.18, SD = 19.29). The QLQ-C30 scale exhibited excellent internal consistency in the present study, as indicated by Cronbach’s alpha coefficient of 0.919 (Table 2).

### 3.3. Associations of Clinical/Demographic Data with the EORTC QLQ-C30 Subscales

**Figure 1:**
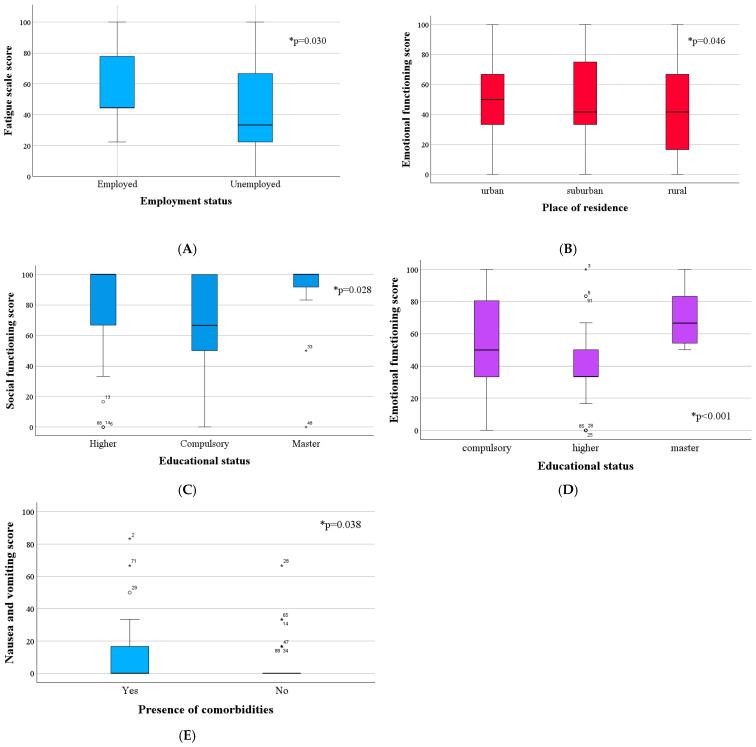
(**A**–**E**) Boxplots illustrate statistically significant associations between demographic and clinical variables with EORTC QLQ-C30 subscale scores in BC patients undergoing chemotherapy. (**A**) Association between employment status and fatigue scores. (**B**) Emotional functioning according to place of residence (urban, suburban, rural). (**C**) Association between educational level and social functioning. (**D**) Emotional functioning according to educational level. (**E**) Nausea and vomiting scores in relation to the presence of comorbidities. Higher scores on functional scales indicate better functioning, whereas higher scores on symptom scales reflect greater symptom burden in the EORTC QLQ-C30. In these plots, the central marker represents the mean value, while the box and whiskers illustrate the variability of the data. Open circles indicate outliers, while star-shaped symbols indicate extreme outliers. Statistical significance between groups was assessed using independent-samples *t*-tests or one-way ANOVA, as appropriate. Asterisks next to *p*-values indicate statistically significant differences between groups (*p* ≤ 0.05).

As illustrated in Figure 1, fatigue represented the most prominent symptom reported by participants, whereas nausea/vomiting and diarrhea demonstrated comparatively lower mean scores. Functional subscales generally showed more favorable scores compared with symptom-related domains. More specifically, statistically significant differences were observed in the mean fatigue scores between employed (M = 57.14, SD = 23.38) and unemployed (M = 43.39, SD = 26.76) BC patients (*p* = 0.030; Figure 1A). Similarly, the mean emotional functioning scores differed significantly by residential location, with urban (M = 52.64, SD = 28.55), suburban (M = 51.73, SD = 27.14), and rural residents (M = 44.75, SD = 32.42) exhibiting distinct outcomes (*p* = 0.045; Figure 1B). Educational status was significantly associated with social functioning scores, with the highest scores observed among participants holding a master’s degree (M = 86.11, SD = 30.84) and the lowest among those with only compulsory education (M = 68.80, SD = 30.15) (*p* < 0.001; Figure 1C). Likewise, emotional functioning scores varied significantly according to educational level, with master’s degree holders reporting the highest mean scores (M = 70.13, SD = 17.92) and individuals with compulsory education reporting the lowest (M = 38.60, SD = 24.23) (*p* < 0.001; Figure 1D). Furthermore, nausea and vomiting scores differed significantly between BC patients with comorbidities (M = 8.46, SD = 2.25) and those without comorbidities (M = 4.81, SD = 1.88) (*p* = 0.038; Figure 1E).

Furthermore, we found statistically significant differences according to the type of treatment given before chemotherapy. Specifically, the analysis showed that BC patients receiving chemotherapy without previous endocrine or radiation therapy demonstrated the highest mean scores in physical (M = 88.14, SD = 10.83) and social functioning (M = 85.18, SD = 22.80), and the lowest levels of fatigue (M = 35.39, SD = 18.49), dyspnea (M = 19.32, SD = 16.04) and financial difficulties caused by physical condition or medical treatment (M = 18.28, SD = 9.87), indicating comparatively better functional status and symptom burden, compared with patients who were treated with radiation therapy. Participants who received endocrine therapy generally showed intermediate outcomes across most functional and symptom domains. Statistically significant differences between groups were observed for physical functioning (F = 3.681, *p* = 0.029), social functioning (F = 3.088, *p* = 0.049), fatigue (F = 7.804, *p* < 0.001), dyspnea (F = 3.561, *p* = 0.032), and financial difficulties caused by physical condition or medical treatment (F = 4.906, *p* = 0.009). These findings highlight the varying impact of different therapeutic modalities on specific dimensions of health-related quality of life in women undergoing chemotherapy for BC (Table 3 and Table 4).

We also found statistically significant differences according to the type of breast surgery. The analysis showed that the type of breast surgery was associated with total QLQ-C30 score (F = 5.205, *p* = 0.007), emotional function (F = 7.545, *p* < 0.001), role function (F = 3.641, *p* = 0.030), social function (F = 3.377, *p* = 0.042) and insomnia (F = 2.954, *p* = 0.035). Women who had undergone mastectomy showed lower total QLQ-C30 (M = 24.16, SD = 22.28), lower emotional function (M = 43.20, SD = 28.43), lower role function (M = 33.33, SD = 29.33), lower social function scores (M = 66.66, SD = 57.73), and higher insomnia (M = 56.41, SD = 20.46) scores, compared to those who had undergone breast conserving surgery or no resection (Table 5 and Table 6).

Finally, we found statistically significant differences according to the time since BC diagnosis. Time since diagnosis was significantly associated with total QLQ-C30 score (F = 6.371, *p* < 0.001), physical functioning (F = 3.687, *p* = 0.028), social functioning (F = 3.251, *p* = 0.043), and financial difficulties caused by physical condition or medical treatment (F = 3.234, *p* = 0.045). Patients with < 3 years since diagnosis reported lower physical (M = 75.13, SD = 22.36) and social functioning (M = 68.91, SD = 36.25). In contrast, patients ≥3 years post diagnosis demonstrated higher total QLQ-C30 scores (M = 40.89, SD = 7.37) and greater financial difficulties caused by physical condition or medical treatment (M = 30.89, SD = 19.81) (Table 7 and Table 8).

### 3.4. Multiple Regression Analysis

Multiple linear regression analysis demonstrated that several demographic and clinical variables were significantly associated with the total QLQ-C30 score. In particular, marital status (*p* < 0.001), place of residence (*p* = 0.001), time since diagnosis (*p* = 0.004), and type of surgery (*p* = 0.010) were significant predictors of HRQoL. Specifically, married patients, those residing in suburban areas, patients diagnosed 1–3 years earlier, and those who had not undergone surgical treatment reported higher overall QLQ-C30 scores compared with the corresponding reference groups. The regression model explained 33.7% of the variance in the total QLQ-C30 score (R^2^ = 0.337, F = 6.001, *p* < 0.001) (Table 9). As concerns multicollinearity, no evidence of problematic multicollinearity was identified across the regression model for the total QLQ-C30 score, with VIF values ranging from 1.024 to 1.235 (mean VIF = 1.089) and tolerance values ranging from 0.810 to 0.976 (mean tolerance = 0.922).

Regarding the functional subscales of the EORTC QLQ-C30, none of the overall regression models reached statistical significance (all *p* > 0.05). While some individual predictors showed nominal statistical significance in specific models, these results should be interpreted with caution due to the lack of overall model significance. Exploratory associations were identified between higher physical functioning and suburban residence (β = 0.257, *p* = 0.011), as well as a shorter duration since breast cancer diagnosis (1–3 years) (β = −0.213, *p* = 0.032), and the absence of prior treatment before chemotherapy (β = −0.199, *p* = 0.015). Additionally, lower Charlson Comorbidity Index (CCI) scores were nominally associated with higher emotional functioning (β = −0.207, *p* = 0.036), while social functioning showed a nominal association with educational level (β = −0.209, *p* = 0.037) (Table 10).

Concerning the EORTC QLQ-C30 symptom subscales, none of the overall regression models reached statistical significance (all *p* > 0.05). The proportion of explained variance ranged from 4.6% (dyspnea) to 15.8% (diarrhea). Even though some individual predictors showed nominal statistical significance in specific models, these findings should be interpreted cautiously, given the lack of overall model significance. Exploratory associations were identified between shorter time since breast cancer diagnosis (1–3 years) and higher pain scores (β = 0.211, *p* = 0.047). Prior treatment before chemotherapy was nominally associated with higher nausea and vomiting scores (β = 0.257, *p* = 0.011), pain (β = 0.209, *p* = 0.036), constipation (β = 0.222, *p* = 0.028), and diarrhea (β = 0.233, *p* = 0.021). Furthermore, the type of surgery showed nominal associations with pain scores (β = −0.237, *p* = 0.026) and financial difficulties related to physical condition or medical treatment (β = −0.249, *p* = 0.024) (Table 11). Based on the above-mentioned, although the multivariable models indicated limited explanatory power for symptom burden, these exploratory findings may indicate potential relationships between selected treatment-related or clinical factors and specific symptom experiences.

Finally, no evidence of problematic multicollinearity was identified across the regression models for the functional and multi-/single-item symptom subscale scores of the QLQ-C30 questionnaire with VIF values ranging from 1.022 to 1.226 (mean VIF score = 1.087) and tolerance values ranging from 0.816 to 0.978 (mean tolerance score = 0.924).

## 4. Discussion

This study aimed to assess HRQoL among women diagnosed with BC in Greece and investigate the impact of selected demographic and clinical factors on physical and social functioning and BC-related symptoms. The findings indicated that women with BC exhibited a moderate level of HRQoL. In other countries, studies have shown that women diagnosed with BC often report moderate-to-low HRQoL, particularly during or shortly after treatment, due to the physical, emotional, and psychosocial burden of the disease [20,21]. In Greece, research on the evaluation of QoL with QLQ-C30 among women with BC remains limited and yields somewhat inconsistent findings, even though Kontodimopoulos et al. [18], confirmed that the Greek version of the QLQ-C30 is a reliable and valid instrument for evaluating HRQoL specifically in Greek BC patients. One of the earliest studies focused exclusively on patients with stage II BC, reporting a decline in QoL during the first year following diagnosis and initial treatment [22]. A subsequent study examined women undergoing active treatment and found QoL to be at a relatively satisfactory level despite ongoing therapy [23]. More recently, Yfantis et al. [24] assessed QoL in women with non-metastatic BC one year post surgery, reporting generally favorable outcomes, with high scores in functional domains and lower in symptoms items. These divergent findings highlight the variability in QoL outcomes depending on disease stage, treatment phase, and timing of assessment. To our knowledge, relatively few studies have evaluated HRQoL specifically among Greek BC patients undergoing active chemotherapy using the EORTC QLQ-C30 scale while simultaneously examining the influence of multiple sociodemographic and clinical factors. Therefore, the present study contributes additional evidence regarding factors associated with HRQoL within the Greek clinical setting and may help inform more individualized supportive care strategies for women with BC.

The present study showed that BC patients receiving chemotherapy reported generally favorable functional outcomes, with high physical and social functioning and moderate emotional, cognitive, and role functioning. However, these outcomes varied according to previous therapy, with patients receiving chemotherapy alone demonstrating significantly better physical and social functioning compared to those who received chemotherapy with prior radiation therapy. We also found that fatigue was the most prominent symptom, while most other symptoms were reported at low levels. Notably, fatigue and dyspnea differed significantly by treatment type, with higher levels observed among women who received multiple treatment modalities. Treatment-related financial difficulties also varied across groups, suggesting that more intensive therapeutic approaches may impose an additional economic burden and inability to work. These findings are partly consistent with previous studies in Greece [23,24] and other countries [25]. In a study conducted in Germany, Pezler et al. [25] reported declines in physical functioning during neoadjuvant chemotherapy, while emotional functioning remained relatively stable, and sleep disturbances were more pronounced than in our study. Similarly, in the USA, Williams et al. [26] identified fatigue as a predominant symptom, with limited impact on physical and social functioning, whereas loss of appetite appeared more frequently in this population. In Ethiopia, Muhamed et al. [27] observed greater impairment in emotional functioning and highlighted substantial financial distress, underscoring the influence of socioeconomic context on QoL outcomes. Increased fatigue and psychological burden during chemotherapy have also been documented in South Korea by Oh et al. [28] and Malaysia by Muthanna et al. [29], reinforcing the central role of fatigue as a determinant of HRQoL. Overall, previous studies highlight the multidimensional nature of QoL during chemotherapy and underscore the need for supportive interventions targeting both symptoms and psychosocial factors.

Furthermore, several sociodemographic and clinical characteristics showed associations with QLQ-C30 outcomes in the univariate analyses conducted in this study. Fatigue was higher among employed patients, while emotional functioning was lower among rural residents. Higher educational level was consistently linked to better social and emotional functioning, whereas nausea and vomiting were more pronounced in patients with comorbidities. These findings are in part consistent with those of Arneja and Brooks [30], who reported better emotional health among BC patients with higher education in Canada, and Yfantis et al. [24], who observed associations between rural residence, financial difficulties, and QoL indicators in Greece, underscoring the influence of contextual and socioeconomic factors on HRQoL in women with BC.

In the present study, univariate analyses suggested associations between time since diagnosis, previous therapy, type of surgery, and several QLQ-C30 outcomes. Patients who previously received other treatment modalities, particularly radiation therapy, reported lower physical and social functioning and higher fatigue levels. Women who had undergone mastectomy reported poorer overall, emotional, role, and social functioning, along with higher insomnia scores, compared with those undergoing breast conserving surgery or no resection. Moreover, patients within the first-year post diagnosis (primary disease phase) tended to report lower physical and social functioning, whereas those at least three years post diagnosis (recurrent disease) reported higher overall QLQ-C30 scores but greater treatment-related financial difficulties. In contrast, in a study conducted in Spain, Miret et al. [31] observed a decline in both functional and symptom domains two years after diagnosis, although emotional functioning and insomnia remained relatively stable. A longitudinal study in Germany further indicated that HRQoL subdimensions vary over time, with some domains improving and others persisting or worsening due to long-term treatment effects [32]. Consistently with our findings, studies from Sweden [29], Germany [30] and South Korea [31] have shown that early-stage or recently diagnosed patients tend to report lower HRQoL, particularly in physical, emotional, and social domains, highlighting the dynamic and time-dependent nature of QoL in BC. Likewise, other studies in Greece also highlight the variability of QoL outcomes: Yfantis et al. [24] reported higher emotional functioning among patients receiving chemotherapy, while reconstructive surgery was linked to improved social functioning; and Goula et al. [33] found better QoL two years post-chemotherapy than during treatment. In contrast, in studies conducted in Saudi Arabia [34,35], and Germany [36], no significant differences in overall QoL across surgery types or treatment modalities were observed. Such inconsistencies likely reflect methodological and population differences, underscoring the complexity of QoL determinants in BC.

In the present study, the multivariable regression analysis for the overall QLQ-C30 score demonstrated statistical significance, indicating moderate explanatory power of the included demographic and clinical variables (R^2^ = 0.337, *p* < 0.001). Specifically, married patients, those residing in suburban areas, patients diagnosed 1–3 years earlier, and those who had not undergone surgical treatment reported higher overall QLQ-C30 scores compared with the corresponding reference groups. Furthermore, suburban residence, shorter time since BC diagnosis (1–3 years), and absence of previous treatment before chemotherapy were all associated with higher physical functioning. In contrast, the multivariable regression models for the functional and symptom HRQoL subscales did not reach overall statistical significance, indicating limited explanatory capacity for these specific domains. Yet, some individual predictors demonstrated nominal associations with selected functional and symptom dimensions and should therefore be interpreted cautiously as exploratory findings. These findings are partly consistent with those reported in Brazil by Campos et al. [37], who identified sociodemographic, clinical, and lifestyle factors as key determinants of post-treatment QoL. Similarly, a study from Germany, by Engel et al. [38], and a study from Poland, by Konieczny et al. [9], emphasized the influence of comorbidities, marital status, and education on functional outcomes. In contrast, Imran et al. [34] reported limited explanatory power of regression models, identifying menopausal status as the sole predictor of physical functioning in BC patients in Saudi Arabia. Such variability across studies underscores the multifactorial and population-specific nature of factors associated with QoL in BC, supporting the need for individualized supportive care strategies.

The main strengths of this study include the evaluation of a homogeneous population of women with BC undergoing active chemotherapy and the simultaneous assessment of multiple sociodemographic and clinical variables potentially associated with HRQoL. Furthermore, by focusing specifically on Greek BC patients during active treatment, the study provides additional insight into HRQoL outcomes within the Greek clinical setting and contributes to a more nuanced understanding of the determinants of QoL among women with BC in Greece. On the other hand, certain limitations should be acknowledged. Firstly, the study was conducted in a single tertiary center and followed a cross-sectional design, which may limit the generalizability of the findings in other populations. Secondly, the absence of longitudinal follow-up precluded evaluation of changes in HRQoL over time and prevented assessment of the long-term impact of treatment on patient-reported outcomes. Thirdly, the relatively small sample sizes within specific treatment subgroups may have reduced the statistical power to detect subtle but clinically meaningful differences. Fourthly, because HRQoL data were collected using self-reported questionnaires, the possibility of reporting bias or subjective response bias cannot be entirely excluded. Still, the multiple univariate comparisons across the various QLQ-C30 subscales may have increased the risk of type I error; therefore, some statistically significant findings should be interpreted with caution, particularly those with marginal *p*-values. Although disease burden may have been partially reflected by variables such as time since diagnosis, detailed tumor staging information was not available and consequently could not be incorporated into the analyses. Since disease stage is a key determinant of treatment selection, symptom burden, prognosis, and HRQoL, the possibility of residual confounding cannot be excluded. In particular, the observed associations between the type of surgery and HRQoL outcomes, including emotional, role, and social functioning, may have been influenced by underlying differences in disease stage, as patients with more advanced disease are more likely to undergo mastectomy and experience greater physical and psychological burden. Likewise, the associations observed between time since diagnosis and HRQoL, as well as those related to previous treatment modalities, may partially reflect differences in disease severity rather than the independent effects of these variables. Thus, our results should be interpreted with caution. Future studies should systematically include detailed oncological characteristics, such as TNM stage, nodal status, metastatic disease, and molecular subtype, to better clarify the independent contributions of disease-related and treatment-related factors to HRQoL.

## 5. Conclusions

In conclusion, the findings of this study indicate that female BC patients undergoing chemotherapy in Greece exhibited a moderate level of HRQoL, which is influenced by several demographic and clinical variables; physical and social functioning were high, with moderate emotional, cognitive, and role functioning. Future research should aim to include larger and more diverse patient cohorts, stratified by treatment modalities and disease stage, to enhance generalizability and analytical precision. Longitudinal designs and integration of biological and psychosocial variables would further support a comprehensive understanding of QoL subdimensions throughout the BC care continuum. These findings highlight the importance of individualized supportive care strategies in order to improve QoL of BC patients.

## Figures and Tables

**Table 1:** Demographic and clinical data of BC patients included in the present study.

BC Patients (*n* = 107)	
Demographic characteristics	
Age (years)	58.11 ± 11.90
Marital status	
Single	29 (27.1%)
Married	49 (45.8%)
Divorced	16 (15.0%)
Widow	13 (12.1%)
Educational level	
Compulsory (primary/secondary) education	55 (51.4%)
Higher (Bachelor’s) degree	39 (36.4%)
Master’s or PhD	13 (12.1%)
Employment status	
Employed	22 (20.6%)
Unemployed	85 (79.4%)
Place of residence	
Urban area	65 (60.7%)
Suburban area	28 (26.2%)
Rural area	14 (13.1%)
Smoking	
Yes	19(17.7%)
No	88 (82.2%)
Clinical characteristics	
Time since BC diagnosis	
<1 year	24 (22.4%)
≥1 year and <3 years	46 (42.9%)
≥3 years	37 (34.5%)
Previous endocrine and/or radiation therapy (before chemotherapy)	
Endocrine therapy only	36 (33.6%)
Radiation therapy only	25 (23.4%)
Both endocrine and radiation therapy	30 (28.0%)
Neither endocrine nor radiation therapy	16 (15.0%)
Surgery	
Mastectomy	65 (60.7%)
Breast conserving surgery	39 (36.4%)
No surgical treatment	3 (2.8%)
Comorbidities	
Yes	46 (42.9%)
No	61 (57.0%
Type of Comorbidity	
Hypertension	38 (35.5%)
Thyroid disease	15 (14.0%)
Diabetes mellitus-Type II	12 (11.2%)
Chronic lung disease	4 (3.7%)
Dyslipidemia	35 (32.7%)
Heart failure	3 (2.8%)
Coronary artery disease	6 (5.6%)
Anemia	9 (8.4%)
Osteoporosis	4 (3.7%)
Gastrointestinal disorders	6 (5.6%)
Other	2 (1.9%)
CCI	7.32 ± 0.94

Note: BC: breast cancer; CCI: Charlson Comorbidity Index. Data are expressed as mean ± standard deviation and as frequencies (n) and percentages (%).

**Table 2:** Reliability and summary results for the subdimensions of the QLQ-C30 scale.

	Mean	SD	Min	Max	95% Confidence Interval (Lower/Upper Bound)	Reliability via Cronbach’s Alpha
Global Health Status/QoL	51.18	19.29	16.67	83.33	47.46/54.89	0.834
Functional scales						
Physical function	80.88	17.65	6.67	100.00	77.52/84.38	0.865
Role function	57.39	25.73	0.00	100.00	52.63/62.60	0.882
Emotional function	51.10	28.80	0.00	100.00	45.11/56.15	0.809
Cognitive function	56.92	27.97	0.00	100.00	51.54/62.42	0.869
Social function	75.63	30.88	0.00	100.00	69.41/81.38	0.831
Symptom scales/items						
Fatigue	46.12	26.60	0.00	100.00	40.76/51.08	0.822
Nausea and vomiting	6.92	15.58	0.00	83.33	3.95/10.01	0.874
Pain	23.11	25.98	0.00	100.00	18.30/28.36	0.853
Dyspnea	21.70	23.92	0.00	100.00	17.27/26.53	0.813
Insomnia	32.70	29.09	0.00	100.00	27.04/38.35	0.882
Loss of appetite	12.89	19.83	0.00	100.00	9.16/16.89	0.806
Constipation	14.15	26.00	0.00	100.00	9.23/19.33	0.811
Diarrhea	3.17	9.83	0.00	33.33	1.27/5.07	0.828
Financial difficulties caused by physical condition or medical therapy	16.35	26.12	0.00	100.00	11.43/21.57	0.807

Note: SD: standard deviation.

**Table 3:** Distribution of total and functional QLQ-C30 scores by type of previous treatment given before chemotherapy.

		M	SD	F	*p*-Value
Total QLQ-C30 score	Endocrine therapy	36.05	11.18	0.437	0.648
	Radiation therapy	35.77	11.23		
	Neither	33.70	9.50		
Functional scales					
Global Health Status/QoL	Endocrine therapy	52.84	21.71	0.923	0.401
	Radiation therapy	47.54	18.52		
	Neither	53.70	15.73		
Physical function	Endocrine therapy	80.15	20.66	3.681	0.029 *
	Radiation therapy	76.27	16.59		
	Neither	88.14	10.83		
Role function	Endocrine therapy	56.43	25.97	2.666	0.074
	Radiation therapy	51.47	26.70		
	Neither	67.28	21.91		
Emotional function	Endocrine therapy	49.81	27.56	0.335	0.716
	Radiation therapy	48.52	32.79		
	Neither	54.63	24.71		
Cognitive function	Endocrine therapy	56.06	28.77	0.415	0.661
	Radiation therapy	54.90	31.39		
	Neither	61.11	22.64		
Social function	Endocrine therapy	75.75	34.56	3.088	0.049 *
	Radiation therapy	67.15	30.01		
	Neither	85.18	22.80		

Note: M: mean; SD: standard deviation. * Significant at *p* < 0.05.

**Table 4:** QLQ-C30 multi- and single-item subscale scores by type of previous treatment given before chemotherapy.

		M	SD	F	*p*-Value
Multi-item symptom scales					
Fatigue	Endocrine therapy	41.91	25.11	7.804	<0.001 *
	Radiation therapy	59.47	29.06		
	Neither	35.39	18.49		
Nausea and vomiting	Endocrine therapy	41.16	11.43	1.579	0.211
	Radiation therapy	50.29	20.10		
	Neither	27.40	13.18		
Pain	Endocrine therapy	28.72	22.09	2.619	0.078
	Radiation therapy	30.39	27.05		
	Neither	15.43	18.44		
Single-item symptom scales					
Dyspnea	Endocrine therapy	25.31	18.93	3.561	0.032 *
	Radiation therapy	30.39	23.73		
	Neither	19.32	16.04		
Insomnia	Endocrine therapy	28.78	26.50	2.173	0.119
	Radiation therapy	41.17	37.65		
	Neither	28.39	17.79		
Loss of appetite	Endocrine therapy	17.52	11.36	0.336	0.716
	Radiation therapy	22.00	14.70		
	Neither	21.20	13.58		
Constipation	Endocrine therapy	26.13	12.87	2.437	0.092
	Radiation therapy	28.28	21.56		
	Neither	21.35	7.40		
Diarrhea	Endocrine therapy	3.78	10.70	0.705	0.496
	Radiation therapy	3.92	10.90		
	Neither	1.23	6.41		
Financial difficulties caused by physical condition or medical treatment	Endocrine therapy	22.83	12.12	4.906	0.009 *
	Radiation therapy	31.21	27.45		
	Neither	18.28	9.87		

Note: M: mean; SD: standard deviation. * Significant at *p* < 0.05.

**Table 5:** Distribution of total and functional QLQ-C30 scores based on the type of breast surgery.

		M	SD	F	*p*-Value
Total QLQ-C30 score	Mastectomy	24.16	22.28	5.205	0.007 *
	Breast conserving surgery	36.40	5.87		
	None	37.59	6.76		
Functional scales					
Global Health Status/QoL	Mastectomy	49.48	19.14	1.032	0.360
	Breast conserving surgery	54.60	19.91		
	None	44.44	9.62		
Physical function	Mastectomy	79.17	18.94	1.334	0.268
	Breast conserving surgery	84.38	14.96		
	None	73.33	17.63		
Role function	Mastectomy	33.33	29.33	3.641	0.030 *
	Breast conserving surgery	64.91	24.44		
	None	54.10	25.17		
Emotional function	Mastectomy	43.20	28.43	7.545	<0.001 *
	Breast conserving surgery	62.50	23.94		
	None	77.77	23.94		
Cognitive function	Mastectomy	54.61	27.71	0.642	0.528
	Breast conserving surgery	60.08	28.61		
	None	66.66	28.86		
Social function	Mastectomy	66.66	57.73	3.377	0.042 *
	Breast conserving surgery	71.02	31.21		
	None	84.21	26.83		

Note: M: mean; SD: standard deviation. * Significant at *p* < 0.05.

**Table 6:** QLQ-C30 multi- and single-item subscale based on the type of breast surgery.

		M	SD	F	*p*-Value
Multi-item symptom scales					
Fatigue	Mastectomy	47.52	27.11	0.366	0.715
	Breast conserving surgery	44.44	25.31		
	None	37.03	39.02		
Nausea and vomiting	Mastectomy	7.94	16.69	0.364	0.696
	Breast conserving surgery	5.26	14.02		
	None	5.55	9.62		
Pain	Mastectomy	25.12	25.87	0.513	0.600
	Breast conserving surgery	19.73	26.52		
	None	22.22	25.45		
Single-item symptom scales					
Dyspnea	Mastectomy	23.58	24.80	0.684	0.507
	Breast conserving surgery	19.29	22.76		
	None	11.11	19.24		
Insomnia	Mastectomy	56.41	20.46	2.954	0.035 *
	Breast conserving surgery	36.31	25.88		
	None	23.33	21.33		
Loss of appetite	Mastectomy	15.38	21.30	1.356	0.262
	Breast conserving surgery	8.77	16.77		
	None	11.11	19.24		
Constipation	Mastectomy	17.94	29.50	1.843	0.164
	Breast conserving surgery	7.89	18.06		
	None	11.11	19.24		
Diarrhea	Mastectomy	4.10	11.03	0.804	0.451
	Breast conserving surgery	1.80	7.64		
	None	0.00	0.00		
Financial difficulties related to physical condition or treatment	Mastectomy	18.46	27.65	0.948	0.391
	Breast conserving surgery	14.03	24.05		
	None	0.00	0.00		

Note: M: mean; SD: standard deviation. * Significant at *p* < 0.05.

**Table 7:** Distribution of total and functional QLQ-C30 scores based on time of diagnosis.

		M	SD	F	*p*-Value
Total QLQ-C30 score	<1 year	20.31	7.61	6.371	<0.001 *
	≥1 year and <3 years	38.44	6.84		
	≥3 years	40.89	7.37		
Functional scales					
Global Health Status/QoL	<1 year	58.69	15.78	2.303	0.105
	≥1 year and <3 years	48.73	18.91		
	≥3 years	49.54	20.96		
Physical function	<1 year	75.13	22.36	3.687	0.028 *
	≥1 year and <3 years	82.46	15.02		
	≥3 years	86.95	10.34		
Role function	<1 year	63.76	21.11	1.489	0.230
	≥1 year and <3 years	58.33	25.76		
	≥3 years	52.25	27.82		
Emotional function	<1 year	55.43	25.29	0.961	0.386
	≥1 year and <3 years	53.07	29.67		
	≥3 years	45.94	29.69		
Cognitive function	<1 year	61.59	24.84	1.371	0.258
	≥1 year and <3 years	59.42	27.36		
	≥3 years	50.90	30.16		
Social function	<1 year	68.91	36.25	3.251	0.043 *
	≥1 year and <3 years	74.27	29.33		
	≥3 years	89.13	19.20		

Note: M: mean; SD: standard deviation. * Significant at *p* < 0.05.

**Table 8:** QLQ-C30 multi- and single-item subscale based on the time of diagnosis.

		M	SD	F	*p*-Value
Multi-item symptom scales					
Fatigue	<1 year	42.02	22.46	0.478	0.621
	≥1 year and <3 years	45.89	24.85		
	≥3 years	48.94	31.03		
Nausea and vomiting	<1 year	5.79	12.91	0.315	0.730
	≥1 year and <3 years	6.15	13.77		
	≥3 years	8.55	19.09		
Pain	<1 year	13.04	15.85	2.478	0.089
	≥1 year and <3 years	27.53	26.10		
	≥3 years	23.87	29.53		
Single-item symptom scales					
Dyspnea	<1 year	15.94	17.02	0.883	0.417
	≥1 year and <3 years	23.91	25.97		
	≥3 years	22.52	24.91		
Insomnia	<1 year	34.78	25.58	0.087	0.916
	≥1 year and <3 years	32.60	31.02		
	≥3 years	31.53	29.34		
Loss of appetite	<1 year	10.14	18.62	0.837	0.436
	≥1 year and <3 years	11.59	17.52		
	≥3 years	16.21	23.06		
Constipation	<1 year	13.04	26.09	1.459	0.237
	≥1 year and <3 years	10.14	22.07		
	≥3 years	19.81	29.87		
Diarrhea	<1 year	5.79	12.91	1.166	0.316
	≥1 year and <3 years	2.89	9.49		
	≥3 years	1.85	7.74		
Financial difficulties caused by physical condition or medical treatment	<1 year	11.47	4.34	3.234	0.045 *
	≥1 year and <3 years	25.89	19.56		
	≥3 years	30.89	19.81		

Note: M: mean; SD: standard deviation. * Significant at *p* < 0.05.

**Table 9:** Multiple regression analysis with total QLQ-C30 score, as a dependent variable.

	Unstandardized Coefficients		Standardized Coefficients	*t*-Test	*p*-Value	95% Confidence Interval for B	
	B	Std. Error	Beta			Lower Bound	Upper Bound
(Constant)	24.112	12.119		1.990	0.049	0.056	48.168
Age (years)	−0.006	0.083	−0.007	−0.074	0.941	−0.170	0.158
Marital status	4.918	1.393	0.320	3.530	<0.001 *	2.153	7.684
Educational status	−1.608	1.050	−0.140	−1.532	0.129	−3.692	0.475
Employment status	2.020	1.500	0.132	1.347	0.181	−0.957	4.996
Place of residence	4.743	1.412	0.309	3.358	0.001 *	1.940	7.547
CCI	0.417	1.070	0.035	0.389	0.698	−1.708	2.542
Time of diagnosis	4.104	1.409	0.284	2.912	0.004 *	1.307	6.902
Type of treatment prior to chemotherapy	−0.906	1.193	−0.068	−0.760	0.449	−3.274	1.461
Type of surgery	3.814	1.453	0.250	2.625	0.010 *	0.932	6.696

R^2^ = 0.337, F = 6.001, *p* < 0.001. Note: CCI: Charlson Comorbidity Index. * Significant at the level of *p* < 0.05.

**Table 10:** Summary of multiple linear regression analysis for independent variables predicting functional sub-scales scores of QLQ-C30 questionnaire.

	Global Health Status/QoL		Physical Functioning		Role Functioning		Emotional Functioning		Cognitive Functioning		Social Functioning	
Variable	Β	*p*-Value	β	*p*-Value	β	*p*-Value	β	*p*-Value	β	*p*-Value	β	*p*-Value
(Constant)		<0.001 *		<0.001 *		<0.001 *		0.021 *		0.007 *		0.023 *
Marital status	−0.146	0.149	0.054	0.445	−0.099	0.326	−0.101	0.201	0.025	0.824	−0.134	0.137
Educational status	−0.034	0.734	0.073	0.467	0.131	0.193	−0.057	0.568	−0.183	0.075	−0.209	0.037 *
Employment status	0.007	0.941	0.086	0.472	−0.076	0.451	−0.020	0.845	0.024	0.812	−0.132	0.192
Place of residence	−0.156	0.116	0.257	0.011 *	−0.120	0.227	0.096	0.371	−0.051	0.621	−0.004	0.965
CCI	0.056	0.576	−0.054	0.785	0.051	0.612	−0.207	0.036 *	−0.093	0.352	−0.092	0.368
Time of diagnosis	−0.195	0.053	−0.213	0.032 *	−0.122	0.224	−0.017	0.864	0.051	0.610	−0.171	0.086
Type treatment before chemotherapy	−0.123	0.256	−0.199	0.015 *	−0.043	0.687	0.028	0.794	−0.046	0.647	−0.048	0.629
Type of surgery	−0.026	0.796	0.154	0.130	0.161	0.108	0.080	0.416	−0.074	0.494	−0.038	0.703
R^2^	0.053		0.626		0.022		0.836		0.119		0.272	
*p*-value	0.103		0.156		0.098		0.126		0.089		0.171	

Note: β: Standardized Beta Coefficients; BC: breast cancer; CCI: Charlson Comorbidity Index. * Significant at the level of *p* < 0.05. Although some individual predictors reached nominal statistical significance, none of the overall regression models achieved statistical significance.

**Table 11:** Summary of multiple linear regression analysis for independent variables predicting multi- and single-item symptoms sub-scales scores of QLQ-C30 questionnaire.

	Fatigue		Nausea and Vomiting		Pain		Dyspnea		Insomnia		Loss of Appetite		Constipation		Diarrhea		Financial Difficulties Caused by Physical Condition or Medical Treatment	
Variable	β	*p*-Value	β	*p*-Value	β	*p*-Value	β	*p*-Value	β	*p*-Value	β	*p*-Value	β	*p*-Value	β	*p*-Value	β	*p*-Value
(Constant)		0.973		0.041 *		0.096		0.883		0.749		0.200		0.983		0.503		0.273
Age (years)	0.063	0.535	0.073	0.463	0.137	0.166	0.022	0.834	0.135	0.192	0.129	0.213	0.021	0.833	−0.011	0.911	0.010	0.923
Marital status	0.048	0.634	−0.093	0.348	−0.042	0.665	−0.038	0.717	0.047	0.649	−0.024	0.815	0.033	0.735	0.137	0.161	0.022	0.826
Educational status	−0.070	0.485	0.112	0.253	−0.073	0.452	−0.028	0.782	0.009	0.929	0.048	0.636	−0.114	0.224	−0.077	0.429	−0.043	0.668
Employment status	0.111	0.265	0.059	0.543	0.119	0.215	0.085	0.402	0.106	0.293	0.032	0.748	0.127	0.191	0.105	0.274	0.113	0.257
Place of residence	−0.085	0.397	−0.004	0.969	0.030	0.757	0.053	0.610	−0.160	0.119	0.088	0.388	0.025	0.797	0.135	0.170	0.024	0.815
CCI	0.157	0.122	0.172	0.083	0.163	0.096	0.041	0.694	−0.051	0.616	0.077	0.453	0.034	0.727	−0.099	0.311	−0.117	0.250
Time of diagnosis	−0.058	0.592	0.173	0.105	0.211	0.047 *	0.010	0.931	0.019	0.862	0.158	0.151	−0.129	0.221	0.107	0.309	0.027	0.803
Treatment before chemotherapy	0.095	0.353	0.257	0.011 *	0.209	0.036 *	0.187	0.076	0.054	0.601	0.171	0.101	0.222	0.028 *	0.233	0.021 *	0.082	0.425
Type of surgery	−0.086	0.428	−0.104	0.327	−0.237	0.026 *	−0.071	0.525	0.022	0.844	−0.103	0.351	−0.196	0.066	0.128	0.226	−0.249	0.024 *
R^2^	0.089		0.134		0.149		0.046		0.060		0.066		0.138		0.158		0.080	
*p*-value	0.425		0.118		0.070		0.867		0.733		0.671		0.100		0.052		0.516	

Note: β: Standardized Beta Coefficients; CCI: Charlson Comorbidity Index. * Significant at the level of *p* < 0.05. Although some individual predictors reached nominal statistical significance, none of the overall regression models achieved overall statistical significance.

## Data Availability

The data presented in this study are available upon request from the corresponding authors. The data is not publicly available due to ethical restrictions.

## Supplementary Materials

The following supporting information can be downloaded at: https://www.mdpi.com/article/10.3390/medicina62061196/s1, Supplementary SA: demographic and clinical information questionnaire (English version); Supplementary SB, Table S1: Descriptive analysis results for each item of the EORTC QLQ-C30 scale; Supplementary SC: signed informed consent form (English version).
