# Supplementary material for: Health-Related Quality of Life in Breast Cancer Patients Undergoing Chemotherapy: A Cross-Sectional Study in Greece
Source: Medicina (Kaunas). 2026 Jun 21;62(6):1196. doi: 10.3390/medicina62061196 (PMC13304299; doi:10.3390/medicina62061196)
Supplement: Supplementary file 1 [file medicina-62-01196-s001.zip › medicina-4211515-supplementary.pdf]

Supplementary Material

Supplementary SA: Demographic and Clinical Information Questionnaire (English Version)

Please complete the following questionnaire. Tick or fill in the appropriate answer. All responses will remain confidential and will be used only for research purposes.

Demographic Information

Age (years): ____________________

Marital status:☐ Single ☐ Married ☐ Divorced ☐ Widowed

Educational level:☐ Compulsory education ☐Bachelor's degree ☐ Master's/PhD

Place of residence:☐ Urban ☐ Suburban ☐ Rural

Smoking status: ☐ Yes ☐ No

Clinical Information

Presence of comorbidities: ☐ Yes ☐ No

If yes, specify: ____

Time since breast cancer diagnosis: ☐<1 year ☐ 1–3 years ☐>3 years

Type of surgery: ☐ Mastectomy ☐ Breast conserving surgery ☐ None

Previous endocrine and/or radiation therapy (before chemotherapy):

☐ Endocrine therapy only ☐ Radiation therapy only ☐ Both ☐ Neither

Supplementary SB, Table S1. Descriptive analysis results for each item of the EORTC QLQ-C30 scale.

	Mean ± sd
1. Do you have any trouble doing strenuous activities, like carrying a heavy shopping bag or a suitcase?	2.2±0.7
2. Do you have any trouble taking a long walk?	1.9±0.9
3. Do you have any trouble taking a short walk outside of the house?	1.3±0.7
4. Do you need to stay in bed or a chair during the day?	1.4±0.7
5. Do you need help with eating, dressing, washing yourself or using the toilet?	1.1±0.3
6. Were you limited in doing either your work or other daily activities?	2.3±0.8
7. Were you limited in pursuing your hobbies or other leisure time activities?	2.2±0.8
8. Were you short of breath?	1.7±0.7
9. Have you had pain?	1.6±0.8
10. Did you need to rest?	2.5±0.9
11. Have you had trouble sleeping?	2.0±0.9
12. Have you felt weak?	2.1±0.9
13. Have you lacked appetite?	1.4±0.6
14. Have you felt nauseated?	1.3±0.6
15. Have you vomited?	1.2±0.4
16. Have you been constipated?	1.4±0.8
17. Have you had diarrhea?	1.1±0.3
18. Were you tired?	2.5±0.9
19. Did pain interfere with your daily activities?	1.8±0.9
20. Have you had difficulty in concentrating on things, like reading a newspaper or watching television?	2.4±1.0
21. Did you feel tense?	2.6±1.0
22. Did you worry?	2.4±1.0
23. Did you feel irritable?	2.6±1.0
24. Did you feel depressed?	2.4±1.0
25. Have you had difficulty remembering things?	2.2±1.0
26. Has your physical condition or medical treatment interfered with your family life?	1.7±0.9

27. Has your physical condition or medical treatment interfered with your social activities?	1.8±1.0
28. Has your physical condition or medical treatment caused you financial difficulties?	1.5±0.8
29. How would you rate your overall health during the past week?	4.0±1.2
30. How would you rate your overall quality of life during the past week?	4.1±1.2

Note: SD: Standard deviation.

Supplementary SC: Signed Informed Consent Form English version)

**CONSENT FORM**<br>**to participate in the research study**	
**Please answer the following questionnaire in order to give written your participation consent to the following study:**	
**Health-Related Quality of Life in Breast Cancer patients undergoing chemotherapy: A cross-sectional study in Greece**	
Question	Yes or No
I have been told that this is an anonymous study that respects participants' identity and clinical data.	
I have received all the appropriate information about this study.	
I have been told that the authors currently plan to submit this study for publication in a medical journal, for educational purposes.	
I am not required to sign this form, and I may refuse to do so.	
I will not be paid in any manner to participate in this study.	
I finally give my permission to participate to the above-mentioned study.	

Surname (just the first letter):		Name:	
Signature:		Date:	
